# Properties of three collagen scaffolds in comparison with native connective tissue: an in-vitro study

**DOI:** 10.1186/s40729-023-00504-z

**Published:** 2023-10-11

**Authors:** Alex Solderer, Nicole Widmer, Andrea Gubler, Kai R. Fischer, Stefan P. Hicklin, Patrick R. Schmidlin

**Affiliations:** Division for Periodontology and Peri-Implant Diseases, Clinic of Conservative and Preventive Dentistry, Center of Dental Medicine, University of Zurich, Plattenstrasse 11, 8032 Zurich, Switzerland

**Keywords:** Collagen matrix, Acellular dermal matrix, Connective tissue graft, Resorption rate, Scanning electron microscope (SEM), Artificial saliva, Simulated body fluid

## Abstract

**Purpose:**

To evaluate collagen scaffolds (CS) in terms of their in vitro resorption behavior, surface structure, swelling behavior, and mechanical properties in physiologically simulated environments, compared with porcine native connective tissue.

**Materials and methods:**

Three test materials—one porcine collagen matrix (p-CM), two acellular dermal matrices (porcine = p-ADM, allogenic = a-ADM)—and porcine native connective tissue (p-CTG) as a control material were examined for resorption in four solutions using a high-precision scale. The solutions were artificial saliva (AS) and simulated body fluid (SBF), both with and without collagenase (0.5 U/ml at 37 °C). In addition, the surface structures of CS were analyzed using a scanning electron microscope (SEM) before and after exposure to AS or SBF. The swelling behavior of CS was evaluated by measuring volume change and liquid absorption capacity in phosphate-buffered saline (PBS). Finally, the mechanical properties of CS and p-CTG were investigated using cyclic compression testing in PBS.

**Results:**

Solutions containing collagenase demonstrated high resorption rates with significant differences (*p* < 0.04) between the tested materials after 4 h, 8 h and 24 h, ranging from 54.1 to 100% after 24 h. SEM images revealed cross-linked collagen structures in all untreated specimens. Unlike a-ADM, the scaffolds of p-CM and p-ADM displayed a flake-like structure. The swelling ratio and fluid absorption capacity per area ranged from 13.4 to 25.5% among the test materials and showed following pattern: p-CM > a-ADM > p-ADM. P-CM exhibited higher elastic properties than p-ADM, whereas a-ADM, like p-CTG, were barely compressible and lost structural integrity under increasing pressure.

**Conclusions and clinical implications:**

Collagen scaffolds vary significantly in their physical properties, such as resorption and swelling behavior and elastic properties, depending on their microstructure and composition. When clinically applied, these differences should be taken into consideration to achieve the desired outcomes.

**Graphical Abstract:**

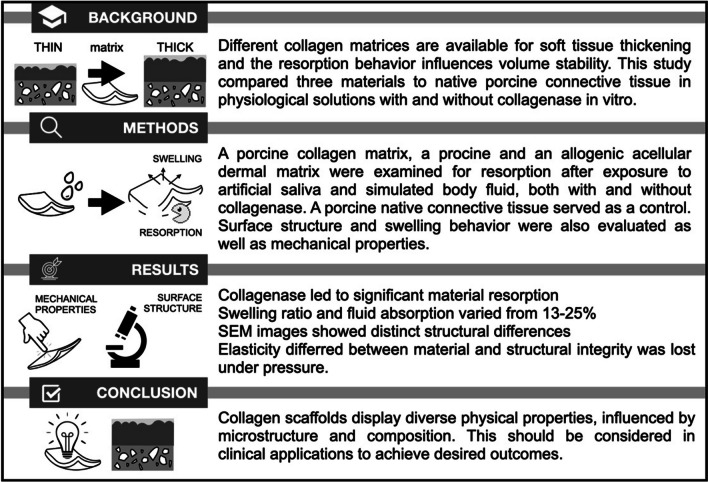

## Background

Trauma or tooth loss typically triggers significant soft and hard tissue remodeling, occasionally leading to severe ridge deficits [1]. To restore an aesthetically pleasing and functionally stable situation after tooth loss, dental implants serve as crucial anchors for fixed and removable dental prostheses [2]. Besides a sufficient bone bed and the so-called “bony envelope” [3], proper soft tissue integration is also essential [4]. This integration is a complex biological process, potentially influenced by various factors [5]. Hence, the conceptual goal is full osseointegration, hemi-desmosomal attachment, and a circular connective tissue cuff around the implant, effectively separating the peri-implant bone from the oral cavity and thus preventing the development of peri-implant inflammation [5]. Avila-Ortiz et al. defined short and long supra-crestal soft tissue height (STH) with a cutoff at 3 mm [6]. Studies have indicated, that a preoperative STH of > 2 mm reduces marginal bone loss around implants [7, 8]. The careful establishment of a stable soft tissue compartment is, therefore, pivotal for the long-term success of dental implants [9]. Thickening thin mucosa results in significantly less crestal bone loss than without soft-tissue thickening [8]. A 2021 consensus paper concluded, “Bone stands hard, but soft tissue is the guard,” emphasizing the bidirectional importance of hard and soft tissues [10].

Furthermore, soft tissues determine the aesthetic success or failure of an implant, particularly in the anterior maxilla. Aesthetic outcomes are achieved with careful soft-tissue handling, which may include the necessity of soft tissue augmentation using autogenous or substitute materials [11].

Considering this, various surgical concepts and materials [12] have been established to thicken soft tissue [12, 13]. At present, autogenous soft tissue grafts—connective tissue grafts (CTG) or free gingival grafts (FGG)—represent the gold standard [2]. However, harvesting soft tissue grafts from the palate might be associated with increased patient morbidity [14]. Moreover, clinicians face challenges with limited standardized availability of autologous tissue quantity and inconsistent quality due to inter-individual anatomical differences in palatal mucosa morphology and anatomical structures, such as vessels and nerves [15, 16]. Treating multiple recessions with autogenous connective tissue is partly restricted due to this limited availability [14]. These factors have led to the development of alternative materials, including acellular dermal matrices (ADM) and collagen matrices (CM) of allogenic or xenogeneic origin. Using these alternatives eliminates the need for an additional surgical site while providing an unlimited supply [17] and potentially shortening surgical time [18]. Although xenogenic CM shows less soft tissue thickening compared to autologous CTG [7], it leads to a reduction of treatment time, postoperative complications, pain, and ultimately results in improved patient satisfaction [13, 19, 20].

However, certain material properties must be met to achieve adequate integration of the biomaterial into the surrounding tissue. An ideal replacement material should be biocompatible and allow rapid cell migration and vascularization [21–25]. Collagen scaffolds (CS) appear to fulfill these requirements, allowing controlled ingrowth of cells and vessels from adjacent tissues [14]. In this process, the original matrix is gradually resorbed and replaced by autogenous tissue [14]. Therefore, the resorption behavior of collagen materials is a relevant parameter for space provision for connective tissue regeneration, ensuring adequate volume stability [21].

Swelling is another crucial parameter. A clinical correlation is believed to exist between a high swelling ratio and rapid resorption rate [26]. After implantation and suturing of the flap, the collagen materials are compressed to varying extents. The pressure remains unpredictable and depends on the flap technique and tissue turgor. In addition, the materials must withstand various forces under physiological (masticatory) loading [21]. Therefore, a certain degree of elasticity may also be advantageous.

In this in-vitro study, three different CS were compared to native porcine CTG in physiological solutions with and without collagenase to simulate biodegradation with respect to various parameters. The objectives were to answer the following questions:

1. What is the resorption capacity of the collagen materials as compared to native porcine connective tissue?
2. How does the morphology of the collagen materials change under the influence of collagenase?
3. What is the swelling capacity of the test materials?
4. How do the CS and the control material behave under mechanical loading?

The null hypotheses were that the materials did not differ concerning the above-mentioned key aspects and parameters.

## Materials and methods

### Materials

In the study, three different CS were examined. Two of these were of porcine origin (p-CM and p-ADM), and one was of human origin (a-ADM). P-CM was a cross-linked collagen matrix (Fibro Gide®, Geistlich Pharma AG, Wollhusen, Switzerland), while p-ADM and a-ADM represented acellular dermal matrices (Mucoderm®, Botiss, Zossen, Germany, and Puros® Dermis Tissue Matrix, Tutogen Medical GmbH, Neunkirchen, Germany). Table 1 provides an overview of the materials used and their properties.

**Table 1:** Overview of the test-materials

Collagen scaffold	Features	Thickness (mm)	Manufacturer
a-ADM = Puros® Dermis Allograft Tissue Matrix	Allogenic origin Sterilization with Tutoplast-procedure Monolayer	ca. 0.8–1.8	Tutogen Medical GmbH; ZimVie
p-CM = Geistlich Fibro-Gide®	Porcine origin Cross-linked Three-dimensional structure	ca. 6.0	Geistlich Pharma AG
p-ADM = Botiss Mucoderm®	Porcine origin Monolayer	ca. 1.2–1.7	Botiss Biomaterials GmbH

Native connective tissue from the porcine palate (p-CTG) served as the control and was stored at − 18 °C. Notably, animals of this study were raised and slaughtered for food production according to the Swiss standards for animal welfare. The study protocol did not in any way influence premortal fate of the animals or the slaughtering process. Therefore, this investigation was not classified as an animal study and the institutional ethics committee did not have any objections to the protocol.

### Solutions

Four reagents were used to test the resorption behavior. These solutions, designed to mimic the oral environment, included simulated body fluid (SBF) and artificial saliva (Klimek), each with and without collagenase (0.5 unit/ml), respectively. SBF and Klimek were prepared exactly according to the instructions provided by Kokubo et al. [27] and Klimek et al. [28], respectively. The ingredients of the used reagents are listed in Tables 3 and 4 in Appendix.

The swelling and cyclic compression tests were conducted in an external laboratory (Geistlich Pharma AG, Wollhusen, Switzerland). This company uses an isotonic phosphate buffered salt solution (PBS) as a reagent for these tests. Therefore, PBS was also employed for these two tests. PBS was prepared according to the company’s recipe.

#### Collagenase

When used clinically, the tested materials undergo natural degradation. To simulate this biodegradation in vitro, collagenase was employed as an enzyme. The collagenase used in this experiment was of bacterial origin, obtained from *Clostridium histolyticum*. Available as a lyophilized powder (C9891, Sigma Aldrich, St. Louis, USA), it was mixed with either SBF or Klimek (5 unit/ml) before being diluted to 0.5 unit/ml.

### Experimental procedure

#### Resorption behavior

Standardized 5 × 5 mm test specimens of the included materials were prepared. The thickness of the samples was measured using an ethanol-purified digital external micrometer (Mitutoyo, Urdorf, Switzerland) to avoid compressing the materials. Five samples (*n* = 5) of each of the four tested materials (three artificial matrices and one p-CTG as control) were exposed to the four solutions at three timepoints (4, 8 and 24 h). Samples were placed in a 24-well plate for 15 min, each in 1 ml of 0.9% sodium chloride solution. After 15 min, the samples were dried and weighed (see “Procedure for standardized drying and weight measurement” section). The sodium chloride solution was removed and replaced by the respective test solutions. At each timepoint, the solutions were exchanged. The 24-well plate containing the samples and replaced solutions was sealed with tape and incubated at 37 °C. At each solution change, CS and control samples were dried and weighed with a Mettler AT261 DeltaRange® scale (Mettler, Greifensee, Switzerland). The resorption rate of the materials was investigated based on the weight measurements.

#### Surface analysis

The surface morphology of the test materials was examined using a scanning electron microscope (SEM; Zeiss, Oberkochen, Germany) at a magnification of 2000×. SEM images were taken in the untreated condition (out of the package) and after 24-h exposure to both SBF with collagenase (0.5 unit/ml) and Klimek with collagenase (0.5 unit/ml), respectively. Before imaging, CS were soaked in deionized water for 3 min and then dried (see “Procedure for standardized drying and weight measurement” section). Subsequently, samples were affixed to an SEM carrier using a self-adhesive carbon pad and sputter-coated with 10 nm of gold.

#### Swelling behavior

**Fig. 1:**
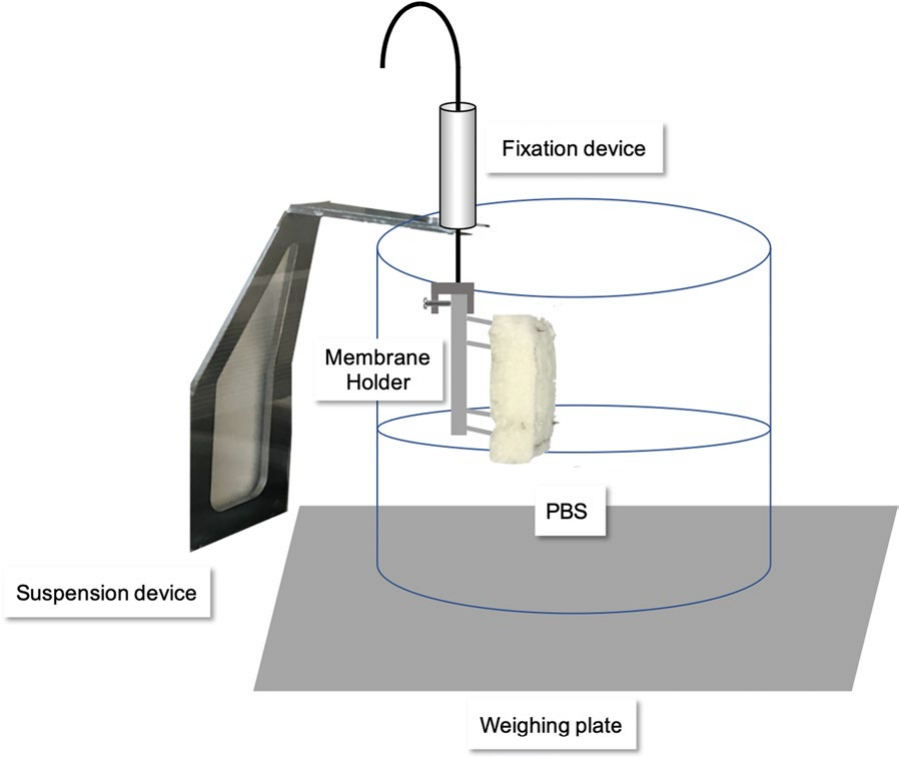
Scheme of the experimental setup

In this experiment, three CS (p-CM, p-ADM, a-ADM) were cut to a size of 15 × 20 mm, with three samples (*n* = 3) of each matrix prepared. Dimensions (length, width and height) were measured at the highest point of the samples using a digital external micrometer (Mitutoyo, Urdorf, Switzerland) at the first visual contact. A pencil line was drawn on each matrix 2 mm from the bottom edge. The prepared samples were vertically mounted on a sample holder attached to a XS204 DeltaRange® scale (Mettler, Greifensee, Switzerland) (Fig. 1). A vessel containing PBS was placed on the balance plate and the bottom 2 mm of each matrix was immersed in PBS. The time and mass change from the immersion of the matrix in the solution until the solution reached the top of the matrix were assessed. Once the matrix appeared fully swollen, the balance plate was lowered, so that the matrix was no longer in contact with the solution. After this, weight change was recorded for an additional 2 min to measure any evaporation. The liquid absorption capacity per volume was calculated by subtracting the mass of the dry matrix from the mass of the swollen matrix and then dividing by the volume of the matrix.

#### Cyclic compression test

The test materials, along with the control material, were cut to a size of 10 × 20 mm. Three specimens (*n* = 3) of each material were prepared. These materials were transferred with forceps into tubes filled with 50 ml of PBS. These tubes were then incubated in a water bath at 37 °C for 2 h. After this 2-h incubation, specimens were placed using bent tweezers under a serrated press plate on the metal plate of the material testing machine (Zwick Roell, Ulm, Germany), fully immersed in PBS at 37 °C. The tool gap was visually adjusted to secure the specimen in place. Cyclic testing (force: 12.1 kPa) of the specimens was performed in PBS at 37 °C for a total of 49 cycles. In the 50th cycle, each specimen was completely pushed through.

#### Procedure for standardized drying and weight measurement

The standardized drying procedure was carried out as follows: at the time of measurement, each sample was removed from the perforated plate using diamond tweezers (Intensiv SA, Collina d’Oro, Switzerland), cleaned with ethanol and placed on a Tela napkin. The napkin was then folded over and weighted down for 5 s with a multi-kilogram lead cylinder. This procedure was repeated on a dry area of the napkin.

Prior to each weight measurement, the scale was calibrated with a plastic pan. Using the diamond tweezers, the specimen was then placed on this pan in the balance for weighing.

### Statistical analysis

The analysis was conducted using the statistical software R. Median, interquartile range (IQR), and the smallest and largest values were calculated from the relative values of the weight changes of the different specimens and compared. Pairwise Wilcoxon Rank Sum Tests were conducted to determine the differences between the materials in the various solutions at the 24-h timepoint. Statistical significance was indicated with an “*” and was always assumed at *p* < 0.05. For the test materials, area, volume change and fluid absorption capacity per volume for each material were calculated. Median, IQR and the smallest and largest values were determined and compared in each case. Pairwise Wilcoxon Rank Sum Tests were used to calculate pairwise comparisons between material levels for swelling behavior. Due to the number of specimens (*n* = 3) no *p* value was obtained.

## Results

### Resorption behavior

**Fig. 2:**
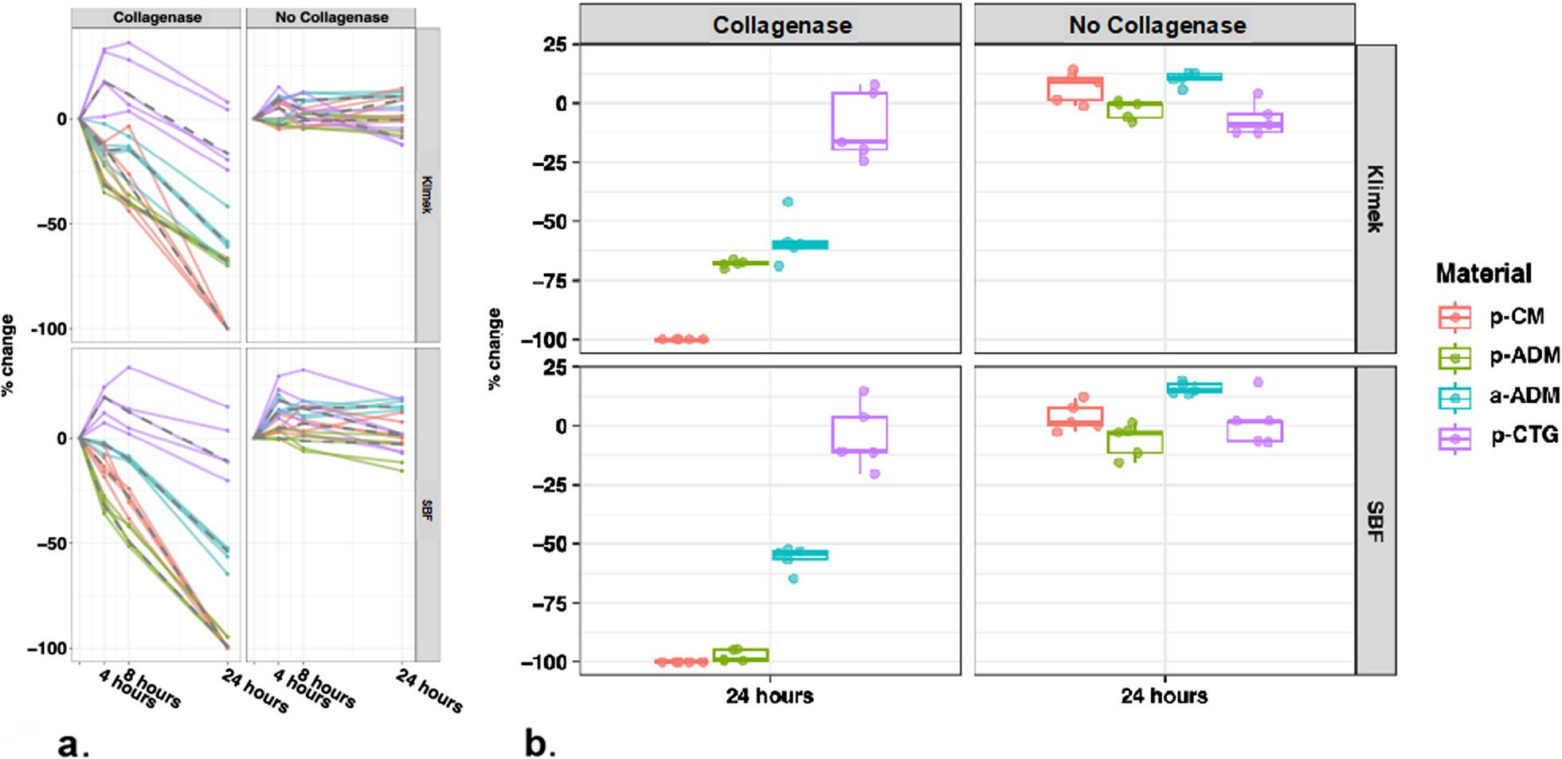
**a** Resorption behavior (%) of included materials over time (h). **b** Box-plots for resorption (%) at 24 h

For the solutions without collagenase, all samples exhibited a comparable and very low to no resorption behavior, with percentage changes ranging from − 6.9 to + 15.8% after 24 h. Please refer to Fig. 2 for more comprehensive information. In contrast, the test samples immersed in the solutions with collagenase demonstrated higher resorption, while the control group (P) exhibited the lowest resorption.

Figure 2 compares the median (IQR) percentage changes in weight for the four investigated materials at different measurement times (4, 8, and 24 h) in both, the SBF collagenase and Klimek collagenase solutions.

In the collagenase-containing solutions, the control material (p-CTG) exhibited weight gain within the first 8 h. However, after 24 h, a weight loss of approximately − 5% was observed in the SBF collagenase solution, and close to − 10% in the Klimek collagenase solution.

#### Comparison of materials in SBF collagenase solution

Comparing resorption values at the measurement timepoint of 24 h, all test and control materials show significant differences (*p* < 0.05) of their resorption behavior in SBF solution with collagenase (Fig. 2).

#### Comparison of materials in Klimek collagenase solution

There is a statistically significant difference between most of the materials in the Klimek collagenase solution (*p* < 0.05). Only between the two acellular dermal matrices (p-ADM, a-ADM), there is no statistically significant difference (*p* > 0.05) of the resorption rate (Fig. 2).

#### Comparison of the materials in collagenase-free solution

No significant difference between the materials in the solutions without collagenase (*p* > 0.05).

### Surface analysis

In the untreated state (original), a-ADM exhibits numerous cross-linked fibers. However, in the SBF collagenase solution, the fine fibers are largely absent, leaving behind primarily thicker fibers. In contrast, the treatment with Klimek collagenase resulted in a distinct redistribution of proteins compared to the SBF collagenase treatment, resulting in a compact appearance of the surface, with the fibers more embedded in the mass.

Upon examination of the untreated state, p-CM reveals a flocculent, multilayered structure at the cut edge. The flakes appear to be cross-linked with each other, although at this low resolution, no definitive conclusion can be drawn regarding molecular cross-linking. In the SEM image of p-CM after treatment with SBF collagenase, salt crystals are visible, which are attributed to a preparation artifact. Similar behavior was observed for p-CM in both the SBF collagenase solution and the Klimek collagenase solution, where the surrounding matrix disintegrated and only remnants of fibers are visible.

SEM images of p-ADM depict a cut surface in its original state. In the untreated state, the structure appears disrupted and flaky, with salt crystals (preparation artifact) observed between the bundles. The structures seem to be strongly cross-linked. After incubation of p-ADM in the SBF collagenase solution, the surface appears highly dense. Similarly, treatment with Klimek collagenase resulted in the loss of the flake structure and densification, leading to a compact and dense surface appearance.

**Fig. 3:**
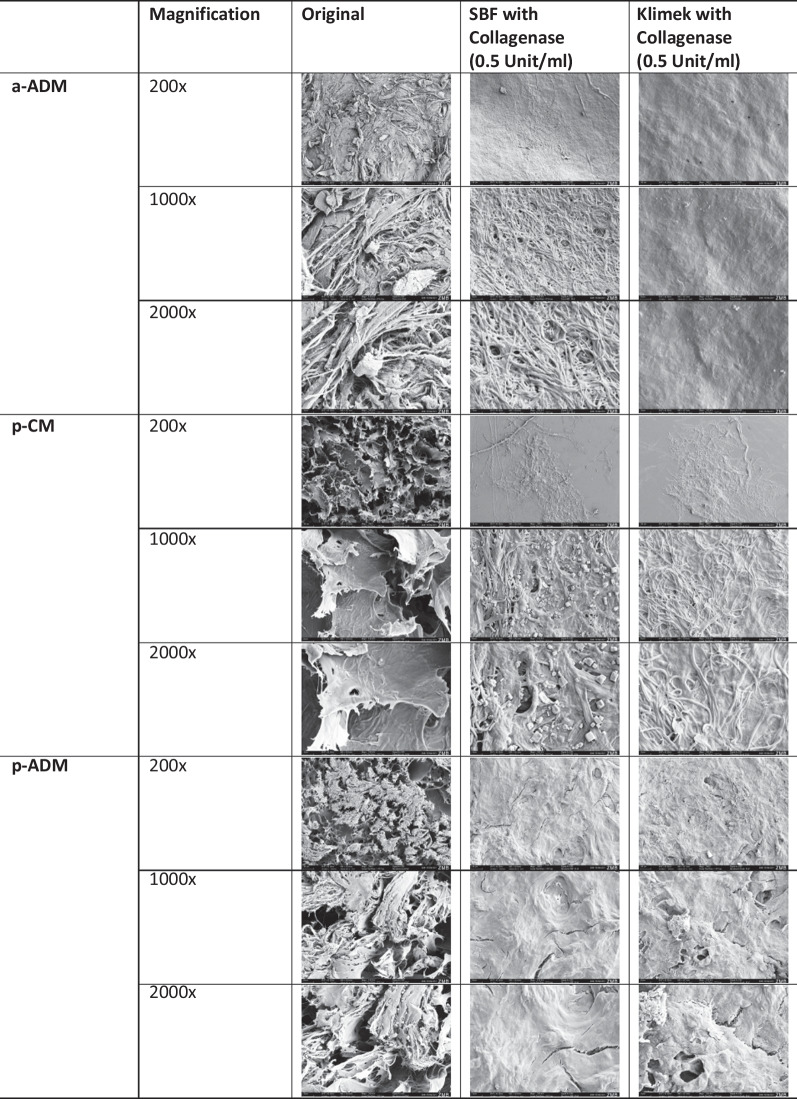
CS at ×2000 magnification in the untreated (original) state, after 24 h incubation in SBF with collagenase and Klimek with collagenase

Figure 3 displays SEM images of CM in the initial state (fresh from packing) and after 24 h of exposure to collagenase solutions (0.5 unit/ml at 37 °C).

### Swelling behavior

Both, area change and volume change (%) were greatest for p-CM, while the two ADM showed less swelling. The trend in terms of volume change was as follows: p-CM > a-ADM > p-ADM.

A similar pattern was observed for the fluid absorption capacity per volume (mg/cm^3^). On median, p-CM exhibited the highest absorption capacity, with the ability to absorb 953.9 mg/cm^3^ of PBS. For p-ADM, this value was 487.8 mg/cm^3^, and for a-ADM, it was 518.3 mg/cm^3^ (Table 2).

**Table 2:** Swelling behavior of test and control materials [area, volume change (%) and PBS uptake (mg/cm^3^)]

	Area change (%)				Volume change (%)				PBS uptake (mg/cm^3^)			
	Median	IQR	Min	Max	Median	IQR	Min	Max	Median	IQR	Min	Max
p-CM	15.0	0.95	13.9	15.8	25.5	0.65	24.8	26.1	953.9	16.9	947.6	981.3
p-ADM	9.1	3.4	5.2	12.0	13.6	2.25	11.0	15.5	487.8	50.8	411.1	512.6
a-ADM	10.1	2.3	9.5	14.1	19.6	3.4	16.3	23.1	518.3	77.2	438.8	593.2

**Fig. 4:**
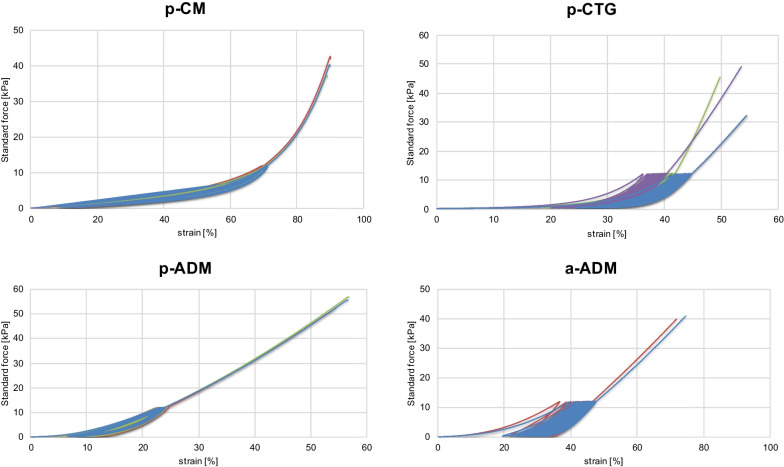
Force–strain diagram for p-CM, p-CTG, p-ADM and a-ADM

Out of three samples of the allogeneic material (a-ADM), only two were evaluable. Figure 4 illustrates the cyclic loading of the four materials. P-CM demonstrates approximately 60% compressibility at a force of 12.1 kPa. The force–strain diagrams of p-CTG and a-ADM exhibit a similar pattern. Both p-CTG and a-ADM experience structural degradation under cyclic loading, resulting in minimal compressibility. On the other hand, p-ADM appears to maintain its structure and exhibit compressibility, although to a significantly lesser extent compared to p-CM. Therefore, p-ADM demonstrates lower elasticity when compared to p-CM.

## Discussion

Autogenous connective tissue grafts and free gingival grafts continue to be the gold standard in periodontal and implant dentistry [12]. Nevertheless, concerns related to patient morbidity, limited supply and inconsistent availability have prompted clinicians to seek alternatives. These alternatives ideally should have similar properties to ensure comparable clinical outcomes to autogenous grafts.

In this study, the authors assessed three different collagen scaffolds regarding their resorptive behavior via weight measurements. We also examined the surface structure of CS using scanning electron microscopy and analyzed swelling behavior and mechanical properties through a cyclic compression test. Native porcine collagen served as the control group.

This research is among the first to examine the resorption behavior and surface morphology of xenogeneic and allogeneic CS in physiological solutions.

The resorption rate results exhibited similar behavior among the tested collagen materials in simulated body fluid (SBF/Klimek, both with and without collagenase). SBF mirrors the ion concentration found in human blood plasma, while Klimek mimics saliva, saturated with calcium and phosphate. Phosphate-buffered saline, used for swelling behavior and cyclic compression testing, is an isotonic solution. All these solutions model a physiological environment in vitro.

In the absence of collagenase, most materials demonstrated a weight increase due to the filling of pores with solution, while a few samples showed a decrease, up to − 6.9%. In the presence of collagenase, the three CS exhibited varying resistance levels. P-CM displayed the lowest resistance, followed by p-ADM, with a-ADM demonstrating the highest. This suggests, that allogeneic ADM might be more collagenase-resistant than the xenogeneic CS. This indicates, that resorption time primarily depends on the CS used. Collagen content and type likely also influence this process [29]. The native control material, p-CTG, showed the highest collagenolytic resistance. These results align with Sbricoli et al., who reported variable resorption times dependent on the CS used [30].

Contradicting these findings, Vallecillo et al. found, that p-CM displayed the greatest resistance to three degradation tests: hydrolytic degradation in phosphate buffer solution, enzyme resistance via white trypsin solution and bacterial collagenase resistance [31]. Our results indicate, that p-CM presented the least resistance in both, SBF and Klimek solutions containing collagenase.

Supporting the previous study, Angele et al. observed, that cross-linking significantly enhanced resistance to enzymatic degradation; a factor potentially influenced by the specific reagents used [32]. Moreover, this study employed a potentially more potent bacterial collagenase (0.5 U/ml), suggesting the rapid degradation of CS might not translate to physiological conditions.

Macroscopically, p-ADM and a-ADM showed a compact structure compared to the sponge-like structure of p-CM. This feature was confirmed by SEM imaging. Cross-linking was achieved with collagen fibers, which could be further investigated by higher magnification SEM imaging. After a 24-h immersion in collagenase-containing solutions, a-ADM and p-ADM underwent densification, suggesting that degradation must precede blood vessel and cell ingrowth in these matrices. In contrast, p-CM lost its flocculent structure after 24 h, which might have initially facilitated fluid penetration and collagenase access, explaining its lower resistance to collagenase-induced resorption.

Rothamel et al. compared cross-linked and native matrices in a canine study. His results revealed, that cross-linked materials exhibited the longest resorption rate but also the highest inflammation rate, potentially complicating wound healing [33]. Thus, less volume-stable native materials may provide the optimal clinical solution.

We also investigated CS swelling behavior. The scaffold of the matrices absorbs liquid, leading to an increase in volume (swelling). The swelling degree and fluid absorption capacity per volume were significantly higher for p-CM than for p-ADM and a-ADM. This could be attributed to p-CM’s sponge-like, multilayered structure, compared to the more compact structure of p-ADM and a-ADM.

In an in vitro test, Zhu et al. observed a correlation between larger pore size and increased liquid absorption capacity and swelling behavior [26]. This could indicate a superior ability to absorb blood and tissue fluid, and thus, endogenous and added exogenous growth factors. However, a high swelling capacity might also lead to a higher rate of wound dehiscences, necessitating more careful flap management by the clinician. Postsurgical wound dehiscences are a common problem, leading to faster resorption of exposed CS and thus less tissue thickening [34].

In our study, while porosity was not directly measured, SEM images revealed a significantly less dense structure in p-CM. This suggests, that p-CM may have a higher porosity than p-ADM and a-ADM. This would explain its superior liquid absorption capacity and swelling behavior.

Finally, we used cyclic compression testing to examine the mechanical properties of the three CS and the control material. P-CM was found to be elastic and compressible, whereas p-ADM showed less elasticity. The allogenic material, a-ADM, displayed little elasticity and lost structural integrity with repeated compression. Interestingly, the native control material, p-CTG, was also found to be minimally compressible and unable to maintain its structural integrity. This could suggest that the allogenic ADM is structurally most like the native material.

The cyclic compression test was performed at 12.1 kPa, a value specifically set by Geistlich Pharma AG (Wollhusen, Switzerland) for p-CM. If a smaller force was applied, it is possible that a-ADM and p-CTG would not lose their structure, yet their elasticity would still be less than that of p-CM.

## Limitations

It is important to acknowledge some limitations in our study setup. This in vitro experiment used a relatively small number of samples (*n* = 5 for resorption behavior experiment, *n* = 3 for swelling behavior and cyclic compression test experiments) at a single center. For future research, we suggest larger, multi-center studies. The results rely on weight measurements and SEM images taken by a single individual, introducing potential for bias. Furthermore, investigator-dependent data collection and lack of information about molecular structure are also limitations. Future research could use techniques, such as 2D gel electrophoresis or higher magnifications for further insights.

Moreover, the use of only three CS in our study may limit the generalizability of our findings. Inclusion of more diverse and newer CS in future studies might yield a broader range of results and more comparative data.

In addition, in this study porcine tissue was utilized as control group. However, it is important to note that pCTG differs from human tissue obtained from the palate in terms of consistency. This discrepancy may have introduced variations in our findings. Moreover, the storage at − 18 °C, while commonly employed, might have affected the tissue’s properties.

Regarding surface morphology, although SEM images provided some understanding of the material structures, a higher magnification and possibly combining SEM with other imaging techniques could provide more detailed information about the surface and internal structures.

It is also noteworthy, that although the use of SBF, Klimek and PBS simulate physiological conditions, they still represent an oversimplified model of the complex biological environment in the human body. In vivo studies or advanced in vitro models, which better simulate the physiological environment and immune responses would provide more clinically relevant results.

In addition, the clinical relevance of mechanical properties should be interpreted with caution. While the cyclic compression test provided useful information on the elasticity and structural integrity of the materials, it does not fully simulate the dynamic and multifactorial mechanical stresses occurring in clinical settings.

Finally, all these findings need to be validated in well-designed clinical studies, which could provide more insight into the clinical performance and patient outcomes associated with these materials. In addition, cost-effectiveness analyses may also be important in guiding the choice of materials in clinical practice.

In conclusion, this research provides valuable insights into the resorption behavior, surface morphology, swelling behavior and mechanical properties of three different CS. Despite its limitations, the study advances our understanding of these properties and their implications for clinical practice in periodontal and implant dentistry. It is hoped that this work will provide a foundation for further studies aimed at optimizing the choice and use of CS in these clinical contexts.

## Conclusions

Within the limitations of the current study, it can be observed that collagen scaffolds vary significantly in their physical properties, such as resorption and swelling behavior and elastic properties, depending on their microstructure and composition. When clinically applied, these differences should be taken into consideration to achieve the desired outcomes.

Collagen scaffolds represent a promising alternative compared to the conventionally applied autogenous connective tissue grafts and should, therefore, be further assessed in future studies.

## Data Availability

All data generated or analyzed during this study are included in this published article.

## Appendix

See Tables 3, 4.

**Table 3:** Composition for 1 L SBF according to Kokubo et al. [27]

Chemicals	Quantity/1 L [g]
Sodiumchloride	8.035
Sodium hydrogen carbonate	0.355
Potassiumchloride	0.225
Di-potassium hydrogen phosphate anhydrous	0.176
Magnesium chloride with 6 water of crystallization	0.311
HCl 1M	39 ml
Calciumchloride mit 2 water of crystallization	0.387
Sodiumsulfate	0.072
Tris(hydroxymethyl)aminomethane	6.118
HCl 1M	0–5 ml

**Table 4:** Composition for 1 L of artificial saliva according to Klimek et al. [28]

Chemicals	Quantity/1 L [g]
Sodiumchloride	0.580
Calciumchloridedihydrate	0.225
Ammoniumchloride	0.160
Potassiumchloride	1.270
Sodiumthiocyanate	0.160
Potassiumdihydrogenphosphate	0.330
Urea	0.200
Di-sodiumhydrogenphosphate	0.340

## Abbreviations

ADM: Acellular dermal matrix
a-ADM: Allogenic acellular dermal matrix
p-ADM: Porcine acellular dermal matrix
AS: Artificial saliva
CM: Collagen matrix
CS: Collagen scaffold
CTG: Connective tissue graft
FGG: Free gingival graft
IQR: Interquartile range
p-CM: Porcine collagen matrix
p-CTG: Porcine connective tissue graft
PBS: Phosphate buffered salt solution
SBF: Simulated body fluid
SEM: Scanning electron microscope
STH: Supracrestal tissue height
